# An Approach to a Comprehensive Test Framework for Analysis and Evaluation of Text Line Segmentation Algorithms

**DOI:** 10.3390/s110908782

**Published:** 2011-09-13

**Authors:** Darko Brodic, Dragan R. Milivojevic, Zoran N. Milivojevic

**Affiliations:** 1 Technical Faculty Bor, V.J. 12, University of Belgrade, 19210 Bor, Serbia; 2 Department of Informatics, Zeleni Bulevar 35, Mining and Metallurgy Institute, 19210 Bor, Serbia; E-Mail: dragan.milivojevic@irmbor.co.rs; 3 Technical College Niš, Aleksandra Medvedeva 20, 18000 Niš, Serbia; E-Mail: zoran.milivojevic@vtsnis.edu.rs

**Keywords:** document image processing, text line segmentation, algorithms, experiments framework, testing, signal detection theory

## Abstract

The paper introduces a testing framework for the evaluation and validation of text line segmentation algorithms. Text line segmentation represents the key action for correct optical character recognition. Many of the tests for the evaluation of text line segmentation algorithms deal with text databases as reference templates. Because of the mismatch, the reliable testing framework is required. Hence, a new approach to a comprehensive experimental framework for the evaluation of text line segmentation algorithms is proposed. It consists of synthetic multi-like text samples and real handwritten text as well. Although the tests are mutually independent, the results are cross-linked. The proposed method can be used for different types of scripts and languages. Furthermore, two different procedures for the evaluation of algorithm efficiency based on the obtained error type classification are proposed. The first is based on the segmentation line error description, while the second one incorporates well-known signal detection theory. Each of them has different capabilities and convenience, but they can be used as supplements to make the evaluation process efficient. Overall the proposed procedure based on the segmentation line error description has some advantages, characterized by five measures that describe measurement procedures.

## 1. Introduction

Text line segmentation is a key step in off-line optical character recognition systems [1]. Any disturbances in this document image processing step will relate to inaccurately segmented text lines. Furthermore, it will result in optical character recognition failure [1].

Text documentation is mainly made up of printed text. It is characterized by well-formed text type which has strong regularity in shape and decent interword and line spacing [2]. Due to these facts text line segmentation of printed documents is a simpler task. Accordingly, techniques for detection of text lines in printed documents are largely successful [3]. On the contrary, text line segmentation of handwritten documents is a complex and diverse problem, complicated by the nature of handwriting, and consequently processing of the handwritten documents has remained a leading challenge in document image processing till now [4].

According to many studies related to the evaluation of algorithms for text parameter extraction, testing is an unavoidable process. Until now, test methods were based mainly on testing algorithms using handwritten or printed text samples obtained from text databases. These testing methods were often accommodated to specific types of scripts and types of algorithms. In addition, the results obtained by different test types were difficult to compare, due to their relative inter-relationships [5].

A new approach to performance evaluation is based on comparing the detected segmentation results with an already annotated ground truth [6]. This approach is called the pixel-based method. Consequently, if the ground-truth line and the corresponding detected line share 90% of pixels this has been claimed as correctly detected lines [7]. However, this is an empirical guideline and cannot distinguish some specific circumstances.

Nevertheless, performance evaluation is a goal-oriented task. This is particularly true for text line segmentation. Few methodologies are established based on this attitude [8–10]. Hence, a similar methodology for the evaluation of algorithms for text segmentation is proposed.

This paper introduces a testing framework for the evaluation of text segmentation algorithms. Some aspects of testing methodology are given in [9]. However, it is based on three synthetic like tests that emulate some of the characteristics of handwritten text. The paper added a handwritten text database as the extension to the previous three tests [10]. It consists of text elements that incorporate mixed text lines, touching components, *etc.* that represent the main challenges in text line segmentation. Furthermore, the proposed experimental framework consists of different types of customizable text patterns as well as handwritten text examples. Namely, each of the given experiments represents a separate entity. In addition, all of the tests can be linked by a bottom-up principle. The method is suitable for different types of letters and languages. Its adaptability is its main advantage.

Furthermore, the evaluation method in [9] relies completely on the *RMSE* methodology. It is extended by the incorporation of the methodology given in [11], which added a new measurement criterion, *SLHR* (Segmentation Line Hit Rate). In this paper, it is redesigned. It introduces a text segmentation error type classification based on five measures. Furthermore, it compares with a binary classification based on three measured experiments [10]. The proposed technique is tested on examples of the water flow algorithm and an algorithm based on the anisotropic Gaussian kernel. Furthermore, both algorithms are compared. Hence, the paper presents an efficient method for the evaluation of text segmentation algorithms.

The paper is organized as follows: in Section 2 the experimental framework for the text line segmentation is presented. Section 3 contains the test evaluation procedure, that involves classification of text objects and text segmentation errors as well as their division according to a binary classification. Section 4 offers a brief introduction to the principle of testing algorithms. Section 5 includes testing results and their evaluation by the proposed methods. Conclusions are given in Section 6.

## 2. Experimental Framework

The evaluation of any text line segmentation algorithm is related to its ability to properly perform text line segmentation. Text line segmentation is performed over different reference samples of text closely related to handwritten text elements, as well as the real ones. The experimental framework for the evaluation of the algorithm’s text line segmentation consists of a few text experiments as follows [9]:

- Multi-line straight text segmentation test,
- Multi-line waved text segmentation test,
- Multi-line fractured text segmentation test,
- Handwritten text segmentation test [10].

**Figure 1.**
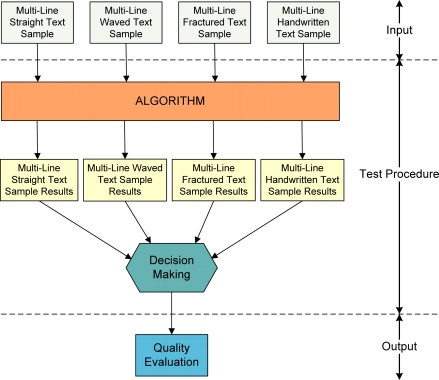
Schematic procedure of the experiments framework.

The overall block diagram of the experimental framework is shown in Figure 1.

The evaluation of the algorithm’s ability to correctly segment text lines is the primary testing role. It is a prerequisite for obtaining other text parameters. Consequently, if the segmentation experiment fails, then further process examination will be meaningless. Hence, its importance is critical.

After the testing process, the obtained results are, in some way, cross-linked. Based on these results, the decision-making process will be achieved. The result of the decision-making procedure is a set of algorithm parameter values. This set is the starting point for the procedure of choosing the algorithm’s optimal parameters.

### 2.1. Multi-Line Straight Text Segmentation Test

**Figure 2.**
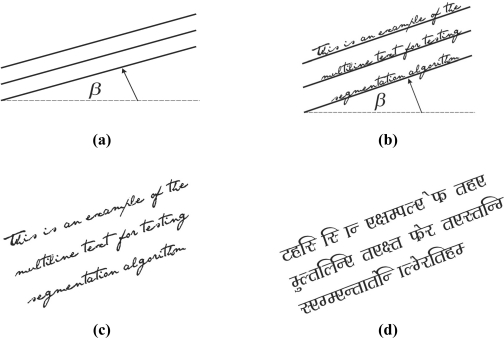
Multi-line straight text: **(a)** Reference line definition. **(b)** Text over reference line. **(c)** English text. **(d)** Bengali text.

The multi-line straight text segmentation test is based on a straight text reference line. Straight text is defined by the skew angle β. Typical values of β that correspond to the handwritten text are those up to 20°. Hence, it takes values from the set {5°, 10°, 15°, 20°} [9]. Furthermore, between line spacing is set to a standard value to 20% of the standard character height [12]. This corresponds to single line spacing. Multi-line straight text samples are shown in Figure 2.

### 2.2. Multi-Line Waved Text Segmentation Test

**Figure 3.**
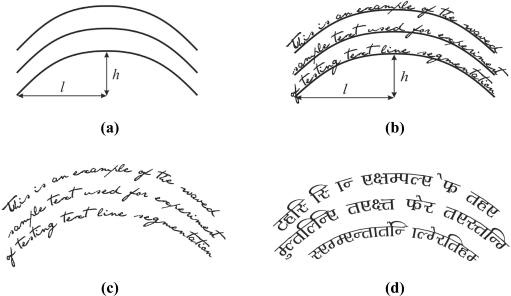
Multi-line waved text: **(a)** Reference line definition. **(b)** Text over reference line. **(c)** English text. **(d)** Bengali text.

The multi-line waved text segmentation test is based on a waved text reference line. Waved text is defined by the parameter ɛ, defined by the expression ɛ = *h*/*l*, where *h* is height, and *l* is half-width of the waved reference line (See Figure 3). Typical values of ɛ that correspond to the previously chosen values of skew angle β are from the set {1/12, 1/6, 1/4, 1/3} [9]. Between line spacing is set to 20% of the standard character height [12]. The resolution of the text samples is 150 and 300 dpi. Multi-line waved text samples are shown in Figure 3.

### 2.3. Multi-Line Fractured Text Segmentation Test

**Figure 4.**
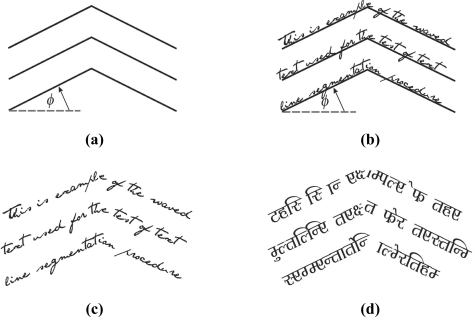
Multi-line fractured text: **(a)** Reference line definition. **(b)** Text over reference line. **(c)** English text. **(d)** Bengali text.

The multi-line fractured text segmentation test is based on a fractured text reference line. Fractured text is defined by the fractured skew angle φ. Typical values of φ that correspond to handwritten text are those up to 20°. Hence, it has values picked from the set {5°, 10°, 15°, 20°} [9]. Furthermore, between line spacing is set to 20% of the standard character height [12]. Resolution of the text samples is 150 and 300 dpi. Multi-line fractured text samples are shown in Figure 4.

### 2.4. Handwritten Text Segmentation Test

**Figure 5.**
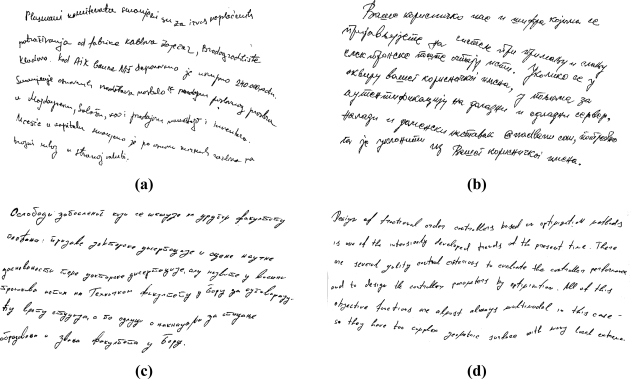
Multi-line handwritten text fragments: **(a)** Serbian Latin text. **(b)** Serbian Cyrillic text. **(c)** Cyrillic text. **(d)** English text.

The multi-line handwritten text segmentation test is based on freestyle handwritten text samples in Serbian Latin, Cyrillic as well as in English scripts [10]. This is a small document text database. The total number of handwritten text samples is 220 text lines. These text samples contain variable skew lines, multi-oriented text as well as mutually inserted words from different text lines. For the sake of conformity, the documents body is the only one considered in the analysis of the text line segmentation. Resolution of the text samples is 150 and 300 dpi. A few handwritten text fragments from the text database are shown in Figure 5.

## 3. Test Results Evaluation

Testing of the algorithm represents the process of applying the algorithm to the proposed text samples. As an implication of the test, the new growing region around the text is raised. The major test assignment is the efficiency evaluation of the text line segmentation process algorithm.

### 3.1. Classification of the Text Objects

It is assumed that during text segmentation a reference sample text containing text objects, called connected-components, is processed by the algorithm. This process leads to a new text object configuration. In an ideal circumstance the number of newly arranged objects corresponds to the correct number of text lines. To make a valid algorithm evaluation the following text elements should be defined [10]:

- Initial objects number *O_init_*,
- Detected objects number *O_det_*, and
- Reference objects number *O_ref_*.

Initial objects *O_init_* represents the starting number of objects in the reference sample text. It is calculated as the counted number of text objects in the starting sample text. After applying the algorithm to the sample text, the number of text objects is changed. Consequently, many text objects are mutually merged by the influence of the text segmentation algorithm. Currently, the number of text objects is given as the number of detected objects *O_det_*. The task of the text segmentation algorithm is to segment text lines hitting or missing this number of lines. Hence, this number of real text lines should be represented as the target number in reference sample text. It is called reference number of objects *O_ref_*. The algorithm efficiency is evaluated by comparing the reference and detected number of objects per each text line.

### 3.2. Classification of the Text Line Segmentation Errors

**Figure 6.**
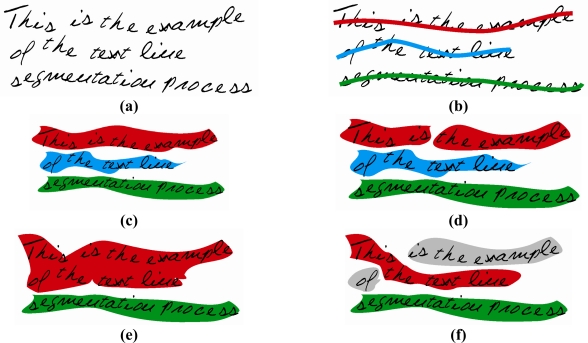
Text line segmentation: **(a)** Original text. **(b)** Original text with reference objects. **(c)** Correctly segmented text lines. **(d)** Over-segmentation text lines. **(e)** Under-segmentation text lines. **(f)** Text lines with mutually inserted words from different text lines.

Text pixels belonging to the initial objects *O_init_* representing the same text line *i* form the reference object *O_ref_* for the line *i*. If the detected object *O_det_* for line *i* is integral and contains objects *O_init_* from the reference object *O_ref_* for the line *i* as subset, then the number of text objects in a distinct text line will be equal to one, which leads to a correctly segmented text line. The number of correctly detected text lines in the sample text is marked as *O_clindet_*. However, all others are defined as error. These circumstances are illustrated in Figure 6.

Segmentation errors are present in the following circumstances:

- Over-segmentation detected text lines *O_ovlindet_* (split lines error *i.e.*, *SLE* [7]),
- Under-segmentation detected text lines *O_unlindet_* (joined lines error, *i.e.*, *JLE* [7]), and
- Detected text lines with mutually inserted words from different text lines *O_mixlindet_* (lines including outlier words, *i.e.*, *LIOW* [7]).

Split lines errors represent the text lines which are wrongly divided by the algorithm into two or more components, *i.e.*, text objects. This circumstance is known as over-segmentation. The joined lines error corresponds to the situation where the sequence of *n* consecutive lines is considered by the algorithm as a unique line. In that case, and if no other error happens, it is considered that one line in the sequence is correct and the other *n*−1 lines of the group are erroneous [7]. This phenomenon is called under-segmentation. Lines including outlier words correspond to lines containing words that are incorrectly assigned to two adjacent lines.

### 3.3. Evaluation of the Algorithm’s Efficiency Based on Errors Type

The algorithm efficiency means the evaluation of the text line segmentation process made by investigated algorithm. If the number of detected objects is closer to the number of reference objects, then the algorithm is more efficient. To evaluate the algorithm’s efficiency the following elements are introduced:

- Segmentation line hit rate, *i.e.*, *SLHR*,
- Over-segmentation line hit rate, *i.e.*, *OSLHR*,
- Under-segmentation line hit rate, *i.e.*, *USLHR*,
- Mixed line hit rate, *i.e.*, *MLHR*, and
- Segmentation root mean square error (*RMSE*), *i.e.*, *RMSE_seg_*.

*SLHR* represents the ratio of the number of correctly segmented text lines over the total number of text lines in the reference sample text. It is defined as:

(1) $$\mathrm{SLHR}=1-\left|\mathrm{RE}\right|=1-\left|\frac{O_{\mathrm{ref}}-O_{\mathrm{clindet}}}{O_{\mathrm{ref}}}\right|$$

Over-segmentation phenomena lead to an increased number of objects per text line. Hence, the boundary growing area created by algorithm hasn’t been successful in merging all objects of the text line into one. As previously stated, the number of the over-segmented lines is marked as *O_ovlindet_*. *OSHLR* represents the ratio of the number of over-segmented text lines over the total number of text lines in the reference sample text. It is defined as:

(2) $$\mathrm{OSLHR}=1-\left|\mathrm{RE}\right|=1-\left|\frac{O_{\mathrm{ref}}-O_{\mathrm{ovlindet}}}{O_{\mathrm{ref}}}\right|$$

The under-segmentation process leads to a smaller number of objects than the number of text lines. Hence, two or more consecutive text lines are considered as a unique one. *USHLR* represents the ratio of the number of under-segmented text lines over the total number of text lines in the reference sample text. It is defined as:

(3) $$\mathrm{USLHR}=1-\left|\mathrm{RE}\right|=1-\left|\frac{O_{\mathrm{ref}}-O_{\mathrm{unlindet}}}{O_{\mathrm{ref}}}\right|$$

The process of mutually injected objects from different text lines leads to mixed text lines. *MLHR* represents the ratio of the number of mixed text lines over the total number of text lines in the reference sample text. It is defined as:

(4) $$\mathrm{MLHR}=1-\left|\mathrm{RE}\right|=1-\left|\frac{O_{\mathrm{ref}}-O_{\mathrm{mixlindet}}}{O_{\mathrm{ref}}}\right|$$

At the end, the number of detected and reference text objects (per each text line) is compared. Hence, the number of reference text objects per line is equal to 1. The variance evaluation is given by the *RMSE* [9]:

(5) $${\mathrm{RMSE}}_{\mathrm{seg}}=\sqrt{\frac{1}{N}\sum_{i=1}^{N}{\left(O_{i,\mathrm{ref}}-O_{i,\mathrm{est}}\right)}^{2}}$$

where *N* is the total number of lines in the reference sample text, *O_i,ref_* is the number of reference objects in the text line *i* (equal to one per each line), and *O*_*i,est* is_ is the number of detected objects in the text line *i*.

### 3.4. Evaluation of the Algorithm’s Efficiency based on Binary Classification

Binary classification is based on the signal detection theory (SDT) postulate [13]. Its task is to classify the members of a given set of objects into two groups, based on whether they have some property or not. Suppose that we test the set of objects for the presence of a property. If some objects have a property and the test confirms it, then those objects are true positives (*TP*) [14]. In an unlikely scenario, some objects do not have a property, but the test confirms it. They are false negatives (*FN*) [14]. Some objects may have the property, but the test mistakenly does not confirm it. These are called false positives (*FP*) [14]. Finally, some objects do not have a property, and the test confirms it. These are true negatives (*TN*) [14]. In the context of classification tasks, the previous statements about the terms true positives, true negatives, false positives and false negatives are used to compare the given classification of an item. This is systemized in Table 1 in the so-called confusion matrix (CM) [14].

From these elements the common evaluation measures can be extracted [14]:

- precision,
- *recall*, and
- *f-measure*.

*Precision* is a measure of the ability of a system to present only relevant items. It is defined as [14]:

(6) $$\mathrm{precision}=\frac{\mathrm{TP}}{\mathrm{TP}+\mathrm{FP}}$$

and it measures the exactness of a classification. A higher *precision* means less false positives, while a lower *precision* means more false positives. This is often at odds with *recall*, as an easy way to improve *precision* is to decrease *recall*.

*Recall* is a measure of the ability of a system to present all relevant items. It is defined as [14]:

(7) $$\mathrm{recall}=\frac{\mathrm{TP}}{\mathrm{TP}+\mathrm{FN}}$$

*Recall* measures the completeness, or sensitivity, of a classifier. Higher *recall* means less false negatives, while lower *recall* means more false negatives. Improving *recall* can often decrease *precision* because it gets increasingly harder to be precise as the sample space increases.

*Precision* and *recall* can be combined to produce a single metric known as *f-measure*, which is the weighted harmonic mean of *precision* and *recall*. It is defined as [14]:

(8) $$f-\mathrm{measure}=2*\frac{\mathrm{precision}*\mathrm{recall}}{\mathrm{precision}+\mathrm{recall}}$$

These elements can be used as common evaluation measures. The following measures are correlated in the text line segmentation [15,16]:

- *TP* represents segmented text line hits *i.e.*, *O_clindet_*,
- *FP* represents segmented text line misses *i.e.*, *O_ovlindet_*, and
- *FN* represents the number of the false segmented text lines *i.e.*, *O_unlindet_* + *O_mixlindet_*.

## 4. Principle of the Testing Algorithm

The smearing method sample for text line segmentation is used. It represents the group of boundary growing algorithms. In smearing methods the consecutive black pixels along the horizontal direction are smeared [17]. The seed points that fulfill predefined criteria activate the process. Consequently, the white space between black pixels is filled with black pixels. It is achieved only if their distance is within a predefined threshold. This way, enlarged areas of black pixels around text are formed. It is so-called boundary growing areas. These areas of the smeared image enclose separated text lines. Hence, obtained areas are mandatory for text line segmentation. In the following text, two testing algorithms will be introduced:

- water flow algorithm, and
- algorithm based on anisotropic Gaussian kernel.

### 4.1. Water Flow Algorithm

**Figure 7.**
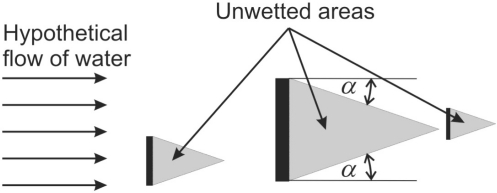
Illustration of the water flow algorithm in direction from left to right (black regions represent text objects *i.e.*, three I letters).

The water flow algorithm proposed in [18] is also used. It will be just briefly explained. The algorithm assumes a hypothetical flow of water in a particular direction across an image frame in such a way that it faces obstruction from the characters of the text lines. As a result of water flow algorithm, unwetted image frames are extracted. These areas represent the triangle shadows that form the so-called unwetted regions. Seed points that activate the algorithm represent the isolated corner points of the text objects. Further, this hypothetical water flow is expected to fill up the gaps between consecutive text lines. Hence, unwetted areas are of major importance for text line segmentation. The circumstance where hypothetical water flows from left to right is shown in Figure 7.

**Figure 8.**
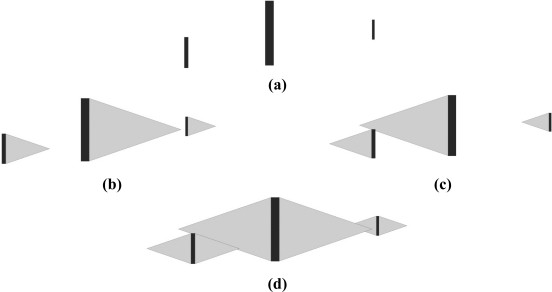
Text line segmentation water flow algorithm involving water flow angle *α*: **(a)** initial text containing three I letters. **(b)** unwetted areas made by water flow from left to right. **(c)** unwetted areas made by water flow from right to left. **(d)** united unwetted areas.

Furthermore, the parameter water flow angle *α* is introduced. It widely affects the unwetted regions shape influencing the text line segmentation process. Hence, the selecting process of the water flow angle value is crucial to the quality of the text line segmentation. The complete process of the water flow algorithm applied on the text sample formed of the three letters I is shown in Figure 8.

**Figure 9.**
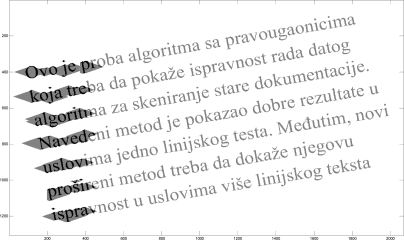
Water flow algorithm applied to the text sample.

Gray regions represent the unwetted areas incorporating initial text objects. The stripes of unwetted areas are labeled for the extraction of text lines. Once the labeling is completed, the image is divided into two different types of stripes. First one contains text lines, while the other one contains line spacing. It is shown in Figure 9.

### 4.2. Algorithm Based on Anisotropic Gaussian Kernel

An algorithm based on the anisotropic Gaussian kernel is also used for testing. It will be explained briefly. Its main principle is expanding black pixel areas of text by scattering every black pixel in its neighborhood. This way, distinct areas that mutually separate text lines are established. Hence, the primary purpose is joining only text elements from the same text line into the same distinct continuous areas. The Gaussian probability function is taken as a template that gives the probability of the random function. Consequently, it represents the probability of the hypothetical expansion around every black pixel representing a text element. Furthermore, around every black pixel, new pixels are non-uniformly dispersed.

**Figure 10.**
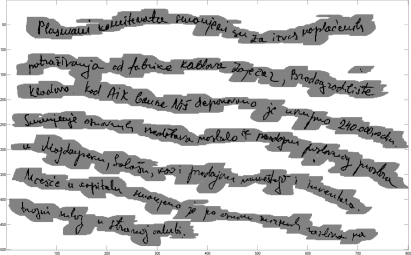
Algorithm based on anisotropic Gaussian kernel applied to the text sample.

These new pixels have lower black intensity. Because the level of probability expansion relates to distance from black pixel, their intensity depends completely on the distance from the original black pixel. However, after applying the Gaussian anisotropic kernel, equal to 2*K* + 1 in the *x*-direction and 2*L* + 1 in the *y*-direction, text is scattered forming an enlarged area around it. Newly created pixels are grayscale. Hence, document text image is a grayscale. Now, inside the kernel a “probability” sub-area is formed using the radius 3*σ_x_* and 3*σ_y_* of ellipse in x and y direction. *σ* represents standard deviation defining curve spread parameter. Converting all these pixels into black pixels as well as inverting image, forms the new black pixel expanded areas [7]. These areas are named boundary-growing areas. The algorithm’s application to the text sample is given in Figure 10.

## 5. Testing and Evaluation

### 5.1. Water Flow Algorithm

For the purpose of testing the algorithm, the parameter water flow angle *α* from the reduced set {10°, 12°, 14°} is used [19,20]. Text samples are converted to 300 dpi resolution. Testing of the algorithm is performed on the example of 96 lines of multi-line straight, waved, and fractured text as well as 220 lines of diverse handwritten text, consisting of a variety of different scripts (over 500 lines of text).

#### 5.1.1. Test Results

The results after applying the algorithm to the four proposed reference text sample groups are presented in Tables 2–5.

#### 5.1.2. Evaluation Based on Error Type

The first evaluation process is based on the text line segmentation error type. The results (from Tables 2–5) are rearranged in the appropriate form validated by measures: *SLHR*, *OSLHR*, *USLHR*, *MLHR*, and *RMSE*. These results are given in Tables 6–9.

The results from the multi-line straight text segmentation test show that there is no mistakenly achieved errors classified as under-segmentation or mixed lines errors. Hence, the only relevant data is received by *SLHR* and *OSLHR*. The choice of water flow angle equal to 10° shows prominently better results. Furthermore, the small *RMSE* value confirms it.

In the multi-line waved text segmentation test the phenomena of under-segmentation appeared. It is raised by decreasing the water flow angle *α*. However, the segmentation line hit rate is improved by reducing *α*. The small value of *RMSE* confirms the advantage of choosing a water flow angle equal to 10°.

In the multi-line fractured text segmentation test decreasing the water flow angle *α* leads to mixed results. Although the segmentation results are slightly better, it shows an increased number of mistakenly recognized lines identified as under-segmented ones. Hence, there is no difference between choosing 10° or 12° for the water flow angle. The similar *RMSE* values reaffirm this.

In the multi-line handwritten text segmentation test use of small water flow angle below 12° noticeably improves the quality of the segmentation process. The *RMSE* value identified this as well.

#### 5.1.3. Evaluation Based on Binary Classification

The evaluation process is based on the binary classification. The results (from Tables 2–5) are rearranged in the appropriate form validated by the measures *precision*, *recall*, and *f-measure*. These results are given in Tables 10–13.

In the multi-line straight text segmentation test, due to the lack of under-segmentation, *precision* is the only relevant measurement element. Hence, the water flow angle election of 10° gives the best results. *F-measure* matched this confirmation.

In multi-line waved text segmentation test, decreasing the water flow angle leads to higher *precision*. However, the occurrence of under-segmentation leads to lower *recall* values. *F-measure* as a combination of *precision* and *recall* illustrates this. Hence, there is no significant advantage between the election of 10° or 12° for the water flow angle.

In the multi-line fractured text segmentation test the results described by *precision* and *recall* are similar for the water flow angle of 10° and 12°. The values of *f-measure* just confirm it.

In the multi-line handwritten text segmentation test the advantage of decreasing the water flow angle is important. Consequently, the *precision* is highly improved. Because under-segmentation elements are missing, the *precision* is the only relevant measure. *F-measure* just follows it.

### 5.2. Algorithm Based on Anisotropic Gaussian Kernel

For the purpose of testing the algorithm based on anisotropic Gaussian kernel, its parameters *K* and *L* are under consideration. The main purpose of testing is the optimization of these parameters. Because of the size of the letters, *K* is picked from the reduced set {5, 8, 10} [12,21]. Furthermore, corresponding the parameter *λ* is used instead of *L*. It is defined as *λ* = *K*/*L*. *λ* is selected from the reduced set {3, 4, 5} [21,22]. All text samples are converted to 300 dpi resolution. Testing of the algorithm is performed on the example of 96 lines of multi-line straight, waved, and fractured text as well as 220 lines of diverse handwritten text, which consist of different variety of scripts (over 500 lines of text).

#### 5.2.1. Test Results

After applying algorithm to the four proposed reference text sample groups, the results obtained are presented in Tables 14–17.

#### 5.2.2. Evaluation Based on Error Type

For the evaluation based on the text line segmentation errors type, results (from Tables 14–17) are rearranged in the appropriate form validated by measures: *SLHR*, *OSLHR*, *USLHR*, *MLHR*, and *RMSE*. These results are given in Tables 18–21.

From the given results, the optimal parameter pairs (*K*, *λ*) are as follows: (5, 4), (5, 5), (8, 3), (8, 4), (8, 5), and (10, 3). Furthermore, the small *RMSE* value (below 0.60) confirms it. It should be noted that enlarging *λ* leads to the under-segmentation phenomena, *i.e.*, to *USLHR* > 0.

From Table 19, *USLHR* and *MLHR* are not expressed. Furthermore, bigger *K* and *λ* lead to better *SLHR*. Hence, the optimal (*K*, *λ*) parameter pairs are as follows: (8, 4), (8, 5), (10, 3), (10, 4), and (10, 5).

In the multi-line fractured text segmentation test, enlarging *K* and *λ* lead to better segmentation results. Although the segmentation results are better, it slightly increases the number of under-segmentation lines. The optimal (*K*, *λ*) parameter pairs are as follows: (8, 4), (8, 5), (10, 3), (10, 4), and (10, 5).

In the multi-line handwritten text segmentation test, use of higher *K* and *λ* improve segmentation results. As a consequence, under-segmentation is more expressed. The optimal (*K*, *λ*) parameter pairs are as follows: (8, 5), (10, 4), and (10, 5). The value of *RMSE* confirms this as well.

#### 5.2.3. Evaluation Based on Binary Classification

The evaluation process is based on the binary classification. The results (from Tables 14–17) are rearranged in the appropriate form validated by measures: *precision*, *recall*, and *f-measure*. These results are given in Tables 22–25.

In the multi-line straight text segmentation test, due to under-segmentation, *recall* is meaningful. Hence, enlarging *K* and *λ* which leads to the under-segmentation, and lower *recall* as well as *f-measure* follows.

In the multi-line waved text segmentation test, good values of *precision* and *recall* are connected with higher *K* and *λ* pairs.

Like to previous test, in the multi-line fractured text segmentation test enlarging the *K* and *λ* pair follows better *precision* and *recall* values.

In the multi-line handwritten text segmentation test the advantage of increasing *K* and *λ* pair is obvious. However, further enlargement of this pair will not afford any improvement of *precision* and *recall*.

### 5.3. Comparative Analysis and Interpretation of the Evaluation Process

The evaluation based on error type contains five distinct measures: *SLHR*, *OSLHR*, *USLHR*, *MLHR*, and *RMSE*. Their interpretation is clear and unmistakable. The fifth measure is *RMSE*, which is clearly distinct in fine tuning segmentation results (See Example #1, and 2 in the Appendix). Obviously, the evaluation based on error type is more clear and remarkable. In contrast, the evaluation based on the binary classification has only three distinct measures: *precision*, *recall*, and *f-measure*. Consequently, the third one is the harmonic mean of the other two. Nevertheless, this evaluation process includes more statistical measures. In [10] evaluation based on binary classification is improved by additional measurement extension. However, both methods have different capabilities and convenience, and they can be used mutually as well. Still, the method with five measures has certain advantages. Hence, it is chosen in the decision-making procedure.

### 5.4. Decision-Making Procedure

From the obtained results, the decision-making procedure is performed. It results as the set of algorithm parameter values, which are the starting point for choosing the algorithm’s optimal parameters. Hence, each test, according to the obtained results, gives the optimal subset of parameter values. These values offer the best response of the algorithm to the specific text samples. Each test experiment is referring as *i*. Furthermore, it means that for the test framework *i* = 1, ..., *N*, where *N* represents the total number of tests. In our case *N* = 4. For each test *i*, the best parameters subset is given as *P_i_*. Finally, the final set of parameters is given as *P_f_*:

(9) $$P_{f}=\cap P_{i}$$

#### 5.4.1. Water Flow Algorithm

For the water flow algorithm comparative results linked with the five measures for different tests are joined in the integral tables e.g., for *SLHR*, *OSHLR*, *USLHR*, *MSLHR* and *RMSE*. From Tables 6–9 the following Tables 26–29 are established.

Results from Tables 26–29 are the key for the decision-making procedure. Consequently, they represent the real picture of the algorithm’s evaluation for text line segmentation. However, Table 30 is linked with the comparative results of *SLHR* in favor of the algorithm parameter *α*.

It is clear that from the test values of parameter *α*, the best response of the algorithm to the various types of text is obtained for the parameter *α* = 10° [20]. In addition, the evaluation of *RMSE* confirms it as well. However, careful examination of the *USLHR* should be taken into consideration for further fine-tuning of the parameter *α*.

#### 5.4.2. Algorithm Based on the Anisotropic Gaussian Kernel

For the algorithm based on the anisotropic Gaussian kernel integral comparative results (see Tables 18–21) concerning *SLHR*, *OSHLR*, *USLHR*, *MSLHR* and *RMSE* are shown in Tables 31–34.

Results from Tables 31–34 are the basis for the procedure of choosing the optimal algorithm parameters. Furthermore, Table 35 is linked with the comparative results of *SLHR* in favor of the algorithm parameters (*K* and *λ*).

Regarding the above results, it is clear that from the testing values of the parameter pair (*K* and *λ*), the best response of the algorithm to the various types of text is obtained for the pair (10, 4) [22]. The *RMSE* evaluation confirms it.

### 5.5. Comparison between Algorithms

The final word in testing efficiency is represented by the comparison of the obtained results between the optimal parameter values of both algorithms. For the water flow algorithm (WF algorithm) the optimal parameter *α* is equal to 10° [20]. Furthermore, for the algorithm based on anisotropic Gaussian kernel (AGK algorithm) the optimal parameter pair is given by the (10, 4). Comparative analysis based on error type classification is given in Tables 36–39.

From Table 36, the WF algorithm affords more uniform *SLHR* results, irrespective of different text types. This is confirmed by better results in the multi-line handwritten text test by a margin of up to 10%.

From Table 37, the AGK algorithm has no problem with over-segmentation phenomena. On the contrary, the WF algorithm has to be improved. However, these circumstances can be overcome by additional morphological post-processing. In addition, in a real situation such as with handwritten text both algorithms are equal.

From Table 38, it is obvious that the AGK algorithm has clear problems with under-segmentation. This is a key which leads to better results of the WF algorithm in a complex and diverse test such as the handwritten text.

**Figure 11.**
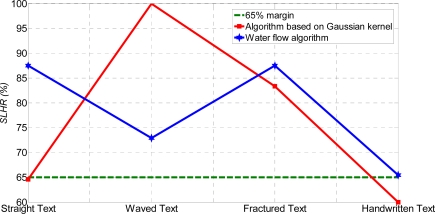
*SLHR* (%) comparison between testing algorithms.

The *RMSE* measure of the WF and AGK algorithms just confirms the previous statements, *i.e.*, the slight advantage of the WF over the AGK algorithm. Figure 11 shows the *SLHR* (%) comparison between the WF and AGK algorithms.

From Figure 11, the WF algorithm can process the various type of text by the *SLHR* margin of over 65%, while the AGK algorithm cannot. Hence, the WF algorithm has a clear advantage over the AGK algorithm. Similar evaluations can be used for the comparison of algorithms by the methodology based on binary classification. However, it has only three measures and some circumstances are not clearly distinct [10] (See Appendix). Furthermore, comparative analysis based on binary classification of errors is given in Tables 40–42.

From Table 40, the AGK algorithm has a clear advantage over WF algorithm in three synthetic-like tests. However, all advantages vanish in a complex test like the multi-line handwritten text.

From Table 41, WS algorithm has more uniform results. Furthermore, this means less under-segmentation elements. Particularly, this is true for multi-line handwritten text testing.

**Figure 12.**
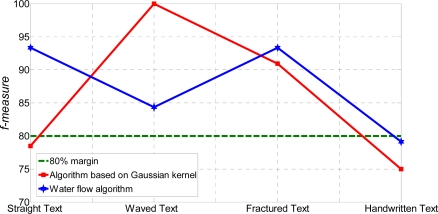
*F-measure* comparison between testing algorithms.

*F-measure* is criteria that reflect all bad and good results of testing. Hence, the evaluation process of the algorithm should be very sensitive to this measure [10]. From Table 42, WF algorithm has been characterized by more uniform level of *f-measure* value. Figure 12 shows *f-measure* comparison between WF and AGK algorithms.

From Figure 12, the WF algorithm can process the various type of text by the *f-measure* margin of around and over 80%, while the AGK algorithm can do so only up to 75%. Again, the WF algorithm has a clear advantage over the AGK algorithm. However, the interpretation process of the binary classification of errors is not so obvious as the error type classification.

## 6. Conclusions

The paper proposes a comprehensive test framework for the evaluation of the algorithms’ effectiveness in the process of text line segmentation. Previously, all testing procedures were custom oriented based on document image databases representing templates. However, the proposed test framework presents a step towards testing generalization in the domain of document image processing algorithms. It consists of four various multi-line text experiments: straight, waved, fractured, and handwritten ones. Further, two suitable validation methods are provided. The first method is based on the text line segmentation error terms. It incorporates five distinct measures. They are inter-related as well. The other one, which is well known and more often used, is based on the binary classification linked with signal detection theory. It consists of three distinct and inter-related measures. Both methods have different capabilities and convenience, but can be used concurrently and supplemented as needed. However, due to the five measures that characterize the measurement process, the method of algorithm evaluation based on error type has certain advantages. In addition, this evaluation process is useful for algorithm assessment as well as for making any conclusions about it. In the end, the adaptability of the comprehensive test framework for different types of letters and languages represents its main advantage.

## Figures and Tables

**Table 1.** Confusion matrix.

Reality on Signal	Yes	No
Present	Hit (*H* or *TP*)	Miss (*M* or *FP*)
Absent	False Alarm (*FA* or *FN*)	Correct Rejection (*CR* or *TN*)

**Table 2.** Multi-line straight text segmentation test results.

***α***	**10°**	**12°**	**14°**
***O_clindet_***	84	68	60
***O_ovlindet_***	12	28	36
***O_unlindet_***	0	0	0
***O_mixlindet_***	0	0	0

**Table 3.** Multi-line waved text segmentation test results.

***α***	**10°**	**12°**	**14°**
***O_clindet_***	70	62	46
***O_ovlindet_***	14	32	50
***O_unlindet_***	12	2	0
***O_mixlindet_***	0	0	0

**Table 4.** Multi-line fractured text segmentation test results.

***α***	**10°**	**12°**	**14°**
***O_clindet_***	84	82	74
***O_ovlindet_***	2	6	20
***O_unlindet_***	10	8	2
***O_mixlindet_***	0	0	0

**Table 5.** Multi-line handwritten text segmentation test results.

***α***	**10°**	**12°**	**14°**
***O_clindet_***	144	96	88
***O_ovlindet_***	76	124	132
***O_unlindet_***	0	0	0
***O_mixlindet_***	0	0	0

**Table 6.** Multi-line straight text segmentation test results.

***α***	**10°**	**12°**	**14°**
***SLHR* (*%*)**	87.50	70.83	62.50
***OSLHR* (*%*)**	12.50	29.17	37.50
***USLHR* (*%*)**	0.00	0.00	0.00
***MLHR* (*%*)**	0.00	0.00	0.00
***RMSE***	0.50	0.65	0.79

**Table 7.** Multi-line waved text segmentation test results.

***α***	**10°**	**12°**	**14°**
***SLHR* (*%*)**	72.92	64.58	47.92
***OSLHR* (*%*)**	14.58	33.33	52.08
***USLHR* (*%*)**	12.50	2.08	0.00
***MLHR* (*%*)**	0.00	0.00	0.00
***RMSE***	0.52	0.78	0.88

**Table 8.** Multi-line fractured text segmentation test results.

***α***	**10°**	**12°**	**14°**
***SLHR* (*%*)**	87.50	85.42	77.08
***OSLHR* (*%*)**	2.08	6.25	20.83
***USLHR* (*%*)**	10.42	8.33	2.08
***MLHR* (*%*)**	0.00	0.00	0.00
***RMSE***	0.35	0.38	0.69

**Table 9.** Multi-line handwritten text segmentation test results.

***α***	**10°**	**12°**	**14°**
***SLHR* (*%*)**	65.45	43.64	40.00
***OSLHR* (*%*)**	34.55	56.36	60.00
***USLHR* (*%*)**	0.00	0.00	0.00
***MLHR* (*%*)**	0.00	0.00	0.00
***RMSE***	0.078	0.141	0.167

**Table 10.** Multi-line straight text segmentation test results.

***α***	**10°**	**12°**	**14°**
***precision* (*%*)**	87.50	70.83	62.50
***recall* (*%*)**	100.00	100.00	100.00
***f-measure* (*%*)**	93.33	82.93	76.92

**Table 11.** Multi-line waved text segmentation test results.

***α***	**10°**	**12°**	**14°**
***precision* (*%*)**	72.92	65.96	47.92
***recall* (*%*)**	85.37	96.88	100.00
***f-measure* (*%*)**	84.34	78.48	64.79

**Table 12.** Multi-line fractured text segmentation test results.

***α***	**10°**	**12°**	**14°**
***precision* (*%*)**	97.67	93.18	78.72
***recall* (*%*)**	89.36	91.11	97.37
***f-measure* (*%*)**	93.33	92.13	87.06

**Table 13.** Multi-line handwritten text segmentation test results.

***α***	**10°**	**12°**	**14°**
***precision* (*%*)**	65.45	43.64	40.00
***recall* (*%*)**	100.00	100.00	100.00
***f-measure* (*%*)**	79.12	60.76	57.14

**Table 14.** Multi-line straight text segmentation test results.

***K***	**5**	**5**	**5**	**8**	**8**	**8**	**10**	**10**	**10**
***λ***	**3**	**4**	**5**	**3**	**4**	**5**	**3**	**4**	**5**
***O_clindet_***	78	88	92	92	82	70	78	62	56
***O_ovlindet_***	18	6	2	0	0	0	0	0	0
***O_unlindet_***	0	2	2	4	14	26	18	34	40
***O_mixlindet_***	0	0	0	0	0	0	0	0	0

**Table 15.** Multi-line waved text segmentation test results.

***K***	**5**	**5**	**5**	**8**	**8**	**8**	**10**	**10**	**10**
***λ***	**3**	**4**	**5**	**3**	**4**	**5**	**3**	**4**	**5**
***O_clindet_***	0	0	6	6	60	92	56	96	96
***O_ovlindet_***	96	96	90	90	36	4	40	0	0
***O_unlindet_***	0	0	0	0	0	0	0	0	0
***O_mixlindet_***	0	0	0	0	0	0	0	0	0

**Table 16.** Multi-line fractured text segmentation test results.

***K***	**5**	**5**	**5**	**8**	**8**	**8**	**10**	**10**	**10**
***λ***	**3**	**4**	**5**	**3**	**4**	**5**	**3**	**4**	**5**
***O_clindet_***	0	0	0	6	72	84	54	80	78
***O_ovlindet_***	94	92	92	86	16	0	32	0	0
***O_unlindet_***	2	4	4	4	8	12	10	16	18
***O_mixlindet_***	0	0	0	0	0	0	0	0	0

**Table 17.** Multi-line handwritten text segmentation test results.

***K***	**5**	**5**	**5**	**8**	**8**	**8**	**10**	**10**	**10**
***λ***	**3**	**4**	**5**	**3**	**4**	**5**	**3**	**4**	**5**
***O_clindet_***	12	24	64	72	88	128	84	132	124
***O_ovlindet_***	208	196	156	148	132	86	136	76	72
***O_unlindet_***	0	0	0	0	0	6	0	12	24
***O_mixlindet_***	0	0	0	0	0	0	0	0	0

**Table 18.** Multi-line straight text segmentation test results.

***K***	**5**	**5**	**5**	**8**	**8**	**8**	**10**	**10**	**10**
***λ***	**3**	**4**	**5**	**3**	**4**	**5**	**3**	**4**	**5**
***SLHR*** (%)	81.25	91.67	95.83	95.83	85.42	72.92	81.25	64.58	58.33
***OSLHR*** (%)	18.75	6.25	2.08	0.00	0.00	0.00	0.00	0.00	0.00
***USLHR*** (%)	0.00	2.08	2.08	4.17	14.58	27.08	18.75	35.42	41.67
***MLHR*** (%)	0.00	0.00	0.00	0.00	0.00	0.00	0.00	0.00	0.00
***RMSE***	0.61	0.29	0.20	0.20	0.38	0.52	0.43	0.60	0.65

**Table 19.** Multi-line waved text segmentation test results.

***K***	**5**	**5**	**5**	**8**	**8**	**8**	**10**	**10**	**10**
***λ***	**3**	**4**	**5**	**3**	**4**	**5**	**3**	**4**	**5**
***SLHR*** (%)	0.00	0.00	6.25	6.25	62.50	95.83	58.33	100.00	100.00
***OSLHR*** (%)	100.00	100.00	93.75	93.75	37.50	4.17	41.67	0.00	0.00
***USLHR*** (%)	0.00	0.00	0.00	0.00	0.00	0.00	0.00	0.00	0.00
***MLHR*** (%)	0.00	0.00	0.00	0.00	0.00	0.00	0.00	0.00	0.00
***RMSE***	3.49	3.11	2.46	2.61	0.66	0.20	0.85	0.00	0.00

**Table 20.** Multi-line fractured text segmentation test results.

***K***	**5**	**5**	**5**	**8**	**8**	**8**	**10**	**10**	**10**
***λ***	**3**	**4**	**5**	**3**	**4**	**5**	**3**	**4**	**5**
***SLHR*** (%)	0.00	0.00	0.00	6.25	75.00	87.50	56.25	83.33	81.25
***OSLHR*** (%)	97.92	95.83	95.83	89.58	16.67	0.00	33.33	0.00	0.00
***USLHR*** (%)	2.08	4.17	4.17	4.17	8.33	12.50	10.42	16.67	18.75
***MLHR*** (%)	0.00	0.00	0.00	0.00	0.00	0.00	0.00	0.00	0.00
***RMSE***	4.07	4.01	3.18	3.42	0.61	0.35	1.34	0.41	0.43

**Table 21.** Multi-line handwritten text segmentation test results.

***K***	**5**	**5**	**5**	**8**	**8**	**8**	**10**	**10**	**10**
***λ***	**3**	**4**	**5**	**3**	**4**	**5**	**3**	**4**	**5**
***SLHR*** (%)	5.45	10.91	29.09	32.73	40.00	58.18	38.18	60.00	56.36
***OSLHR*** (%)	94.55	89.09	70.91	67.27	60.00	39.09	61.82	34.55	32.73
***USLHR*** (%)	0.00	0.00	0.00	0.00	0.00	2.73	0.00	5.45	10.91
***MLHR*** (%)	0.00	0.00	0.00	0.00	0.00	0.00	0.00	0.00	0.00
***RMSE***	0.763	0.442	0.266	0.237	0.178	0.118	0.202	0.102	0.125

**Table 22.** Multi-line straight text segmentation test results.

***K***	**5**	**5**	**5**	**8**	**8**	**8**	**10**	**10**	**10**
***λ***	**3**	**4**	**5**	**3**	**4**	**5**	**3**	**4**	**5**
***precision* (%)**	81.25	93.62	97.87	100.00	100.00	100.00	100.00	100.00	100.00
***recall* (%)**	100.00	97.78	97.87	95.83	85.42	72.92	81.25	64.58	58.33
***f-measure* (%)**	89.66	95.65	97.87	97.87	92.13	84.34	89.66	78.48	73.68

**Table 23.** Multi-line waved text segmentation test results.

***K***	**5**	**5**	**5**	**8**	**8**	**8**	**10**	**10**	**10**
***λ***	**3**	**4**	**5**	**3**	**4**	**5**	**3**	**4**	**5**
***precision* (%)**	0.00	0.00	6.25	6.25	62.50	95.83	58.33	100.00	100.00
***recall* (%)**	−	−	100.00	100.00	100.00	100.00	100.00	100.00	100.00
***f-measure* (%)**	−	−	11.76	11.76	76.92	97.87	73.68	100.00	100.00

**Table 24.** Multi-line fractured text segmentation test results.

***K***	**5**	**5**	**5**	**8**	**8**	**8**	**10**	**10**	**10**
***λ***	**3**	**4**	**5**	**3**	**4**	**5**	**3**	**4**	**5**
***precision* (%)**	0.00	0.00	0.00	6.52	81.82	100.00	62.79	100.00	100.00
***recall* (%)**	0.00	0.00	0.00	60.00	90.00	87.50	84.38	83.33	81.25
***f-measure* (%)**	−	−	−	11.76	85.71	93.33	72.00	90.91	89.66

**Table 25.** Multi-line handwritten text segmentation test results.

***K***	**5**	**5**	**5**	**8**	**8**	**8**	**10**	**10**	**10**
***λ***	**3**	**4**	**5**	**3**	**4**	**5**	**3**	**4**	**5**
***precision* (%)**	5.45	10.91	29.09	32.73	40.00	59.81	38.18	63.46	63.27
***recall* (%)**	100.00	100.00	100.00	100.00	100.00	95.52	100.00	91.67	83.78
***f-measure* (%)**	10.34	19.67	45.07	49.32	57.14	73.56	55.26	75.00	72.09

**Table 26.** Comparative results for *SLHR* (%) measurement (*α* is the algorithm parameter).

***α***	**Multi-line straight text**	**Multi-line waved text**	**Multi-line fractured text**	**Multi-line handwritten text**
**10°**	87.50	72.92	87.50	65.45
**12°**	70.83	64.58	85.42	43.64
**14°**	62.50	47.92	77.08	40.00

**Table 27.** Comparative results for *OSLHR* (%) measurement (*α* is the algorithm parameter).

***α***	**Multi-line straight text**	**Multi-line waved text**	**Multi-line fractured text**	**Multi-line handwritten text**
**10°**	12.50	14.58	2.08	34.55
**12°**	29.17	33.33	6.25	56.36
**14°**	37.50	52.08	20.83	60.00

**Table 28.** Comparative results for *USLHR* (%) measurement (*α* is the algorithm parameter).

***α***	**Multi-line straight text**	**Multi-line waved text**	**Multi-line fractured text**	**Multi-line handwritten text**
**10°**	0.00	12.50	10.42	0.00
**12°**	0.00	2.08	8.33	0.00
**14°**	0.00	0.00	2.08	0.00

**Table 29.** Comparative results for *RMSE* measurement (*α* is the algorithm parameter).

***α***	**Multi-line straight text**	**Multi-line waved text**	**Multi-line fractured text**	**Multi-line handwritten text**
**10°**	0.50	0.52	0.35	0.08
**12°**	0.65	0.78	0.38	0.14
**14°**	0.79	0.88	0.69	0.17

**Table 30.** Comparative results for *SLHR* (%) in favor of *α*.

***SLHR(α)***	**Multi-line straight text**	**Multi-line waved text**	**Multi-line fractured text**	**Multi-line handwritten text**
**>50%**	≤14°	≤12°	≤14°	<12°
**>60%**	≤14°	≤12°	≤14°	<12°
**>70%**	≤12°	≤10°	≤14°	-
**>80%**	≤10°	−	≤12°	-
**>90%**	−	−	−	-

**Table 31.** Comparative results for *SLHR* (%) measurement (*K* and *λ* are the parameter pair).

***K, λ***	**Multi-line straight text**	**Multi-line waved text**	**Multi-line fractured text**	**Multi-line handwritten text**
**8, 4**	85.42	62.50	75.00	40.00
**8, 5**	72.92	95.83	87.50	58.18
**10, 4**	64.58	100.00	83.33	60.00
**10, 5**	58.33	100.00	81.25	56.36

**Table 32.** Comparative results for *OSLHR* (%) measurement (*K* and *λ* are the parameter pair).

***K, λ***	**Multi-line straight text**	**Multi-line waved text**	**Multi-line fractured text**	**Multi-line handwritten text**
**8, 4**	0.00	37.50	16.67	60.00
**8, 5**	0.00	4.17	0.00	39.09
**10, 4**	0.00	0.00	0.00	34.55
**10, 5**	0.00	0.00	0.00	32.73

**Table 33.** Comparative results for *USLHR* (%) measurement (*K* and *λ* are the parameter pair).

***K, λ***	**Multi-line straight text**	**Multi-line waved text**	**Multi-line fractured text**	**Multi-line handwritten text**
**8, 4**	14.58	0.00	8.33	0.00
**8, 5**	27.08	0.00	12.50	2.73
**10, 4**	35.42	0.00	16.67	5.45
**10, 5**	41.67	0.00	18.75	10.91

**Table 34.** Comparative results for *RMSE* measurement (*K* and *λ* are the parameter pair).

***K, λ***	**Multi-line straight text**	**Multi-line waved text**	**Multi-line fractured text**	**Multi-line handwritten text**
**8, 4**	0.38	0.66	0.61	0.178
**8, 5**	0.52	0.20	0.35	0.118
**10, 4**	0.60	0.00	0.41	0.102
**10, 5**	0.65	0.00	0.43	0.125

**Table 35.** Comparative results for *SLHR* (%) in favor of pair (*K*, *λ*).

***SLHR(K, λ)***	**Multi-line straight text**	**Multi-line waved text**	**Multi-line fractured text**	**Multi-line handwritten text**
**>50%**	(8,4), (8,5), (10,4), (10,5)	(8,4), (8,5), (10,4), (10,5)	(8,4), (8,5), (10,4), (10,5)	(8,5), (10,4), (10,5)
**>60%**	(8,4), (8,5), (10,4)	(8,4), (8,5), (10,4), (10,5)	(8,4), (8,5), (10,4), (10,5)	(10,4)
**>70%**	(8,4), (8,5)	(8,5), (10,4), (10,5)	(8,4), (8,5), (10,4), (10,5)	−
**>80%**	(8,4)	(8,5), (10,4), (10,5)	(8,5), (10,4), (10,5)	−
**>90%**	−	(8,5), (10,4), (10,5)	−	−

**Table 36.** Comparative algorithms results for *SLHR* (%) measure.

**Algorithm**	**Multi-line straight text**	**Multi-line waved text**	**Multi-line fractured text**	**Multi-line handwritten text**
**WF**	87.50	72.92	87.50	65.45
**AGK**	64.58	100.00	83.33	60.00

**Table 37.** Comparative algorithms results for *OSLHR* (%) measure.

**Algorithm**	**Multi-line straight text**	**Multi-line waved text**	**Multi-line fractured text**	**Multi-line handwritten text**
**WF**	12.50	14.58	2.08	34.55
**AGK**	0.00	0.00	0.00	34.55

**Table 38.** Comparative algorithms results for *USLHR* (%) measure.

**Algorithm**	**Multi-line straight text**	**Multi-line waved text**	**Multi-line fractured text**	**Multi-line handwritten text**
**WF**	0.00	12.50	10.42	0.00
**AGK**	35.42	0.00	16.67	5.45

**Table 39.** Comparative algorithms results for *RMSE* measure.

**Algorithm**	**Multi-line straight text**	**Multi-line waved text**	**Multi-line fractured text**	**Multi-line handwritten text**
**WF**	0.50	0.52	0.35	0.08
**AGK**	0.60	0.00	0.41	0.102

**Table 40.** Comparative algorithms results for *precision*.

**Algorithm**	**Multi-line straight text**	**Multi-line waved text**	**Multi-line fractured text**	**Multi-line handwritten text**
**WF**	87.50	72.92	92.67	65.45
**AGK**	100.00	100.00	100.00	63.46

**Table 41.** Comparative algorithms results for *recall*.

**Algorithm**	**Multi-line straight text**	**Multi-line waved text**	**Multi-line fractured text**	**Multi-line handwritten text**
**WF**	**100.00**	**85.37**	**89.36**	**100.00**
**AGK**	**64.58**	**100.00**	**83.33**	**91.67**

**Table 42.** Comparative algorithms results for *f-measure*.

**Algorithm**	**Multi-line straight text**	**Multi-line waved text**	**Multi-line fractured text**	**Multi-line handwritten text**
**WF**	**93.33**	**84.34**	**93.33**	**79.12**
**AGK**	**78.48**	**100.00**	**90.91**	**75.00**

**Figure A1.**
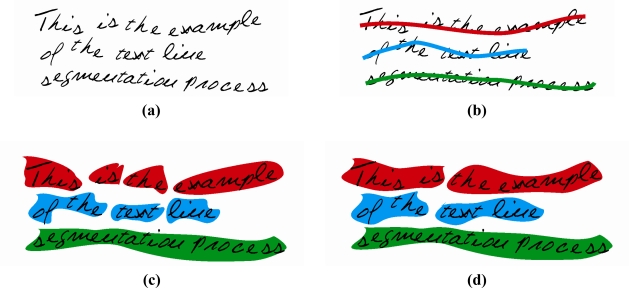
Text line segmentation: **(a)** Original text. **(b)** Original text with reference objects. **(c)** Text after segmentation made by the algorithm #1. **(d)** Text after segmentation made by the algorithm #2.

**Figure A2.**
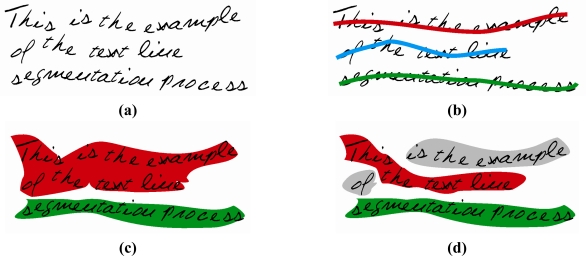
Text line segmentation: **(a)** Original text. **(b)** Original text with reference objects. **(c)** Text after segmentation made by the algorithm #1. **(d)** Text after segmentation made by the algorithm #2.

## Appendix

To understand clearly the purpose of the *RMSE* measurement two different segmentation results are evaluated by it.

### 1. Example #1

After the process of text line segmentation by the algorithms #1 and #2, obtained results are shown in Figure A1.

From Figure A1(c), the results are as follows:

- ***O_clindet_*** = 1,
- ***O_ovlindet_*** = 2, and
- ***O_unlindet_*** = ***O_mixlindet_*** = 0.

Consequently, the number of objects per over-segmented line is:

- Line #1 = 4,
- Line #2 = 3, and
- Line #3 = 1.

From Figure A1(d), the results are as follows:

- ***O_clindet_*** = 1,
- ***O_ovlindet_*** = 2, and
- ***O_unlindet_*** = ***O_mixlindet_*** = 0.

Furthermore, the number of objects per over-segmented lines is:

- Line #1 = 2,
- Line #2 = 2, and
- Line #3 = 1.

### 2. Evaluation of the Algorithm’s Efficiency Based on Error Type (Example #1)

All test results from algorithm #1 and #2 are reorganized according to segmentation error type. The results are presented in Table A1.

**Table A1.** Text line segmentation test results (Example #1).

***Algorithm***	***Algorithm #1***	***Algorithm #2***
***SLHR* (%)**	33.33	33.33
***OSLHR* (%)**	66.66	66.66
***USLHR* (%)**	0.00	0.00
***MLHR* (%)**	0.00	0.00
***RMSE***	1.20	0.47

### 3. Evaluation Based on Binary Classification (Example #1)

All test results from algorithm #1 and #2 are reorganized according to segmentation binary classification. The results are presented in Table A2.

**Table A2.** Text line segmentation test results (Example #1).

***Algorithm***	***Algorithm #1***	***Algorithm #2***
***Precision* (%)**	33.33	33.33
***Recall* (%)**	100.00	100.00
***F-measure* (%)**	50.00	50.00

According to *RMSE*, the algorithm #2 shows slightly better performances than algorithm #1 in the domain of text line segmentation.

### 4. Example #2

After the process of text line segmentation by the algorithms #1 and #2, obtained results are shown in Figure A2.

From Figure A2(c), the results are as follows:

- ***O_ovlindet_*** = 0,
- ***O_mixlindet_*** = 0,
- ***n*** (representing the sequence of consecutive lines) = 2 [11]. Hence, it follows ***O_unlindet_*** = 1, *i.e.*, (*n*−1) [11], and
- ***O_clindet_*** = 1 + 1 = 2 [11].

From Figure A2(d), the results are as follows:

- ***O_clindet_*** = 1;
- ***O_ovlindet_*** = 0,
- ***O_unlindet_*** = 0, and
- ***O_mixlindet_*** = 2.

### 5. Evaluation of the Algorithm’s Efficiency Based on Error Type (Example #2)

All test results from algorithm #1 and #2 are reorganized according to segmentation errors type. The results are presented in Table A3.

**Table A3.** Text line segmentation test results (Example #2).

***Algorithm***	***Algorithm #1***	***Algorithm #2***
***SLHR* (%)**	66.66	33.33
***OSLHR* (%)**	0.00	0.00
***USLHR* (%)**	33.33	0.0
***MLHR* (%)**	0.00	66.66
***RMSE***	0.58	0.82

Accordingly, *MLHR* represents the most penalized error due to the difficult process of identification and correction.

### 6. Evaluation Based on Binary Classification (Example #2)

All test results from algorithm #1 and #2 are reorganized according to segmentation binary classification. The results are presented in Table A4.

**Table A4.** Text line segmentation test results (Example #2).

***Algorithm***	***Algorithm #1***	***Algorithm #2***
***Precision* (%)**	100.00	100.00
***Recall* (%)**	66.66	33.33
***F-measure* (%)**	80.00	50.00

From, Table A4 the evaluation based on binary classification penalizes all errors equivalently. However, evaluation of the algorithm’s efficiency based on error type makes a distinction among different error types (See Table A3), which explains its clear advantage. According to *RMSE*, the algorithm #2 show slightly better performance than algorithm #1 in the domain of text line segmentation.
